# Photographic Reproduction and Enhancement Using HVS-Based Modified Histogram Equalization

**DOI:** 10.3390/s21124136

**Published:** 2021-06-16

**Authors:** Yung-Yao Chen, Kai-Lung Hua, Yun-Chen Tsai, Jun-Hua Wu

**Affiliations:** 1 Department of Electronic and Computer Engineering, National Taiwan University of Science and Technology, Taipei 106, Taiwan; 2 Department of Computer Science and Information Engineering, National Taiwan University of Science and Technology, Taipei 106, Taiwan; hua@mail.ntust.edu.tw; 3 Graduate Institute of Automation Technology, National Taipei University of Technology, Taipei 106, Taiwan; t106618009@ntut.edu.tw (Y.-C.T.); t108618018@ntut.edu.tw (J.-H.W.)

**Keywords:** intelligent vision sensing, photographic reproduction, human visual system, image enhancement, histogram equalization

## Abstract

Photographic reproduction and enhancement is challenging because it requires the preservation of all the visual information during the compression of the dynamic range of the input image. This paper presents a cascaded-architecture-type reproduction method that can simultaneously enhance local details and retain the naturalness of original global contrast. In the pre-processing stage, in addition to using a multiscale detail injection scheme to enhance the local details, the Stevens effect is considered for adapting different luminance levels and normally compressing the global feature. We propose a modified histogram equalization method in the reproduction stage, where individual histogram bin widths are first adjusted according to the property of overall image content. In addition, the human visual system (HVS) is considered so that a luminance-aware threshold can be used to control the maximum permissible width of each bin. Then, the global tone is modified by performing histogram equalization on the output modified histogram. Experimental results indicate that the proposed method can outperform the five state-of-the-art methods in terms of visual comparisons and several objective image quality evaluations.

## 1. Introduction

The human visual system (HVS) is a delicate and complex system. To perceive real-world scenes, human eyes function as visual sensors to receive lights reflected from the surface of objects. Light enters the cornea and refracts; the amount of light entering is regulated by the iris by adjusting the size of the pupil. Then, the ciliary muscle changes the shape of the lens to make the light focus on the retina, where photoreceptors convert the light into electrical signals. Finally, these signals are transmitted to the brain and interpreted as visual images.

Modern people only need to take out their mobile phones from their pockets to capture memorable moments. However, before the camera was invented, people could only record the scenes they saw through words and paintings. As early as the middle of the sixteenth century, inventors began studying imaging technology to lay the foundation for the development of cameras. At the end of the nineteenth century, the Eastman Kodak Company produced film negatives and gradually popularized cameras, and in 1975, they designed the first digital camera that captured a real-world scene by using electronic photodetectors and stored it as a digitized file.

Since the invention of digital cameras, digital photography has evolved rapidly, and people’s requirements for image quality are getting higher and higher. Currently, some people choose to use high-dynamic-range (HDR) sensors to record brightness information transmitted in the real world. HDR images use a 32-bit floating-point format to record details and natural tones in a scene. However, although HDR camera technology is mature, it is limited by the technology of traditional displays. Although screen manufacturers have introduced HDR displays, their prices are too high for them to find widespread use; therefore, low-dynamic-range (LDR) or standard-dynamic-range (SDR) screens that can only display 256 brightness levels are still popular. Therefore, many studies are being conducted to develop photographic tone reproduction methods or tone mapping methods to address this problem. In addition, some interesting works on histogram and image enhancement are recently proposed [1,2,3,4,5,6]. In this paper, we present a novel reproduction method for the conversion of HDR to LDR images that can enhance local details and maintain global naturalness.

## 2. Related Works and Research Motivation

Currently, most photographic reproduction methods can be classified into three categories: global-based, local-based, and hybrid-based methods. Global-based photographic reproduction methods employ the typical mapping strategies, such as linear mapping, exponential mapping, and logarithmic mapping. To upgrade the quality of the subjective viewing experience, Lenzen and Christmann [7] focused on improving the contrast rather than improving the brightness because they thought the most essential part of reproduction is to increase global contrast. Jung and Xu [8] enhanced the overall contrast of the image by using a transfer function called perceptual quantization, which is based on the human contrast sensitivity that represents the human visual perception of luminance. Khan et al. [9] used an HVS-based optimization step to identify pixels in the histogram bins that are indistinguishable to the human eye and then combined the original histogram and the reconstructed histogram to create a new one for designing the mapping curve. Because the shape of the retinal response curve is asymmetric, Lee et al. [10] used the zone system (a classic photography technique) to obtain a new type of asymmetric sigmoid curve (ASC). By using ASC, the curvature of mapping curves can be determined, and the global contrasts of LDR images can be expanded.

Local-based photographic reproduction methods yield suitable transfer functions for individual pixels. Gu et al. [11] proposed three assumptions and designed a local edge-preserving filter that avoids gradient reversal to perform multiscale decomposition of images. Barai et al. [12] integrated a saliency map with the edge-preserving guided filter and also enhanced the detail layer that is rich in edge information. Then, they used HVS-based parameters to adjust both the saturation and the exposure. Mezeni et al. [13] focused on maximizing the available dynamic range. They performed tone compression in the logarithm domain to reduce drastic changes in the dynamic range. Then, in order to modify the appearances of the tone-mapped results, tone compression in the linear domain was also performed. Reproduction methods based on the gradient domain have also been developed. Fattal et al. [14] presented a reproduction method in which the degree of compression is increased as the gradient becomes larger. Their assumption was that by considering the gradient, the fine details could be preserved as the dynamic range is compressed drastically. Mantiuk et al. [15] also proposed a gradient-based method to enhance the contrast and maintain the polarity of the local contrast (i.e., avoid the artificial artifacts caused by gradient reversal) by imposing additional constraints during the gradient process. Unlike global-based methods, local-based methods tend to focus on adjusting the local contrast by considering adjacent pixels. Although details are thus preserved effectively, there is a high probability of generating artificial artifacts, especially for those pixels at salient edges.

In light of the disadvantages of using global- or local-based reproduction methods alone, studies are increasingly combining the properties of these two in hybrid frameworks. Most hybrid-based reproduction methods can be divided into two different types: cascaded architecture and parallel architecture.

In cascaded-architecture-type hybrid reproduction methods, global and local processes are connected in series. Reinhard et al. [16] applied traditional photography schemes to digital images. To overcome the dynamic issue, they proposed a dodging-and-burning technique; however, it tends to generate artifacts such as halos. Ferradans et al. [17] proposed a reproduction method that considers the characteristic of cones (i.e., photoreceptor cells) in the first global stage; in the subsequent stage, the loss of visual contrast was compensated locally. Although they tried to manipulate the saturation perceived by human eyes, the tones of resultant images were not sufficiently vivid. Benzi et al. [18] presented a hybrid reproduction method that reproduces the adaptation mechanism in the retina. They proposed a virtual retina model that takes pupil adaptation into account; unfortunately, some images tended to have a gray-like appearance.

In parallel-architecture-type hybrid reproduction methods, the modular technique is usually used to subdivide the framework into many small units that can be applied independently. Input images are substituted into different modules so that their characteristics can be considered from different aspects through a weighted fusion. Raffin et al. [19] presented a parallel-based method that uses a tone reproduction curve and a local contrast expansion scheme for detail-rich areas. Artusi et al. [20] applied local mapping at regions with high frequencies and a global mapping at the remaining regions. However, the rendered image may be unsatisfactory in some cases, especially in the boundary between locally and globally tone-mapped regions. Yang et al. [21] applied adaptively generated gamma curves to regions with different brightness levels and then performed adaptive weight fusion. The tone-mapped results successfully render a balanced tone between lightness and darkness but tended to lose details. Miao et al. [22] presented a hybrid framework containing two parallel models, where the macro-model manipulates contrasts and the micro-model adjusts details. Although the global information is obtained adaptively, the tones of the resultant images are somehow blurred because of the final fusion process.

**Figure 1:**
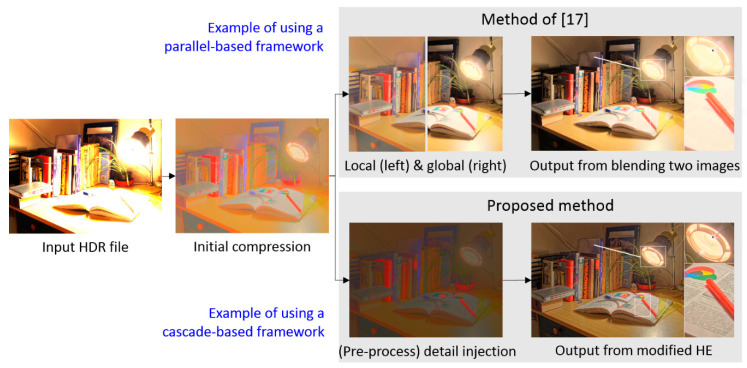
Preliminary comparison between parallel-based (**top**) and cascade-based (**bottom**) photographic reproduction methods, which illustrates the motivation of this study. In the proposed method, we utilized the HVS-based modified histogram equalization (HE) to avoid the fusion loss from blending two images, which was the main reason why we adopted the cascaded-architecture-type hybrid reproduction strategy. Detailed comparisons are provided in Section 4.

- *Motivation for this study*: Recently, the hybrid-based approach seems to be a promising solution to the photographic reproduction problem. However, as mentioned in the above two paragraphs, there is still room for improvement. As shown in Figure 1, the algorithm of [23] presents a typical parallel-architecture-type hybrid reproduction framework, in which the image information content is used to separately enhance each pixel in global contrast and in local details to different extents, following which a weighted fusion is performed. However, if the tone reproduction process involves this type of parallel architecture and fusion, the resultant images might bias to one of the global and local characteristics. Consequently, the parallel-architecture-based method sacrifices either the global tone naturalness or the local details more or less.
- *Contribution of this study*: In view of the shortcoming of the parallel-architecture-based method, this work presents a cascaded-architecture-type reproduction method. Despite having the advantage of computational efficiency, photographic reproduction methods using a monotonic transfer function are typically vulnerable to detail loss (i.e., loss of the local features), especially in the bright and dark areas. In this study, we demonstrate a practical reproduction method and demonstrate that even though it applies the monotonic transfer function (i.e., the proposed HVS-based modified histogram equalization), it is able to preserve the global contrast and even enhance the local details in bright and dark areas simultaneously. To adopt the histogram equalization scheme in photographic reproduction, the histogram configuration is reallocated according to two HVS characteristics: the just noticeable difference and the threshold versus intensity curve. The experimental results demonstrate the effectiveness of the proposed method in terms of different evaluations.

## 3. Proposed Approach

**Figure 2:**
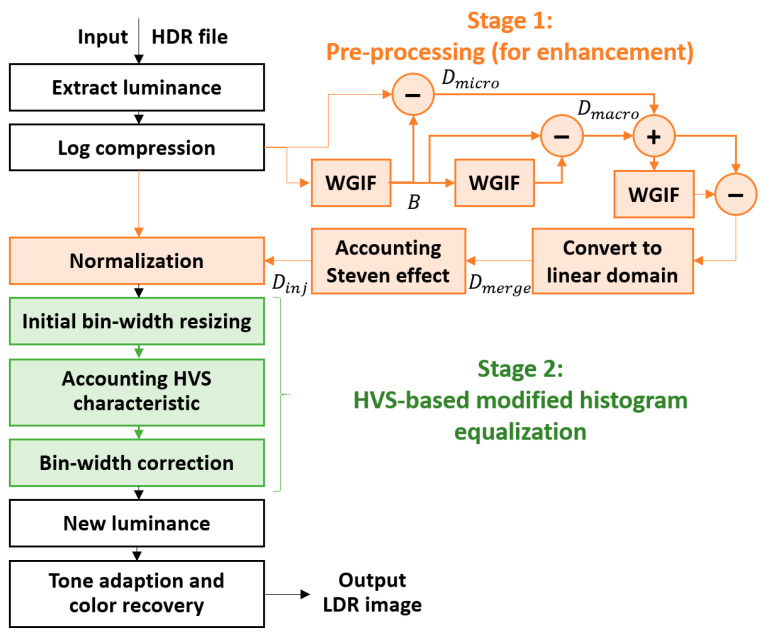
The overall framework of the proposed cascaded-architecture-type reproduction method, where WGIF indicates the weighted guided image filtering technique [24]. In this paper, two stages were designed to complement each other to achieve the advantages of both the local and the global operators.

Figure 2 shows the overall framework of the proposed reproduction method. Unlike in the case of the parallel-architecture-based reproduction method, we prioritized regional features to preserve as much detail as possible in the first stage. This strategy may cause concerns over sacrificing the global tone; however, because the human eye is only sensitive to the regional contrast (i.e., distinguishing between relative bright and dark) and not to the absolute value of the luminance difference [14], we believed that retaining the regional characteristics of the image was more important than rendering a natural global tone. Therefore, in the first stage of the proposed method, we expand the local contrast of the input image by enhancing the local features. In the second stage, the dynamic range is allocated according to the composition of the entire image and the properties of the HVS to recover the natural tone adaptively. As a result, the re-rendered image is closer to the real scene, and the high contrast and regional details are maintained. We believe that the two stages of the proposed method can complement each other so that the advantages of both the local and the global operators can be achieved.

### 3.1. Luminance Extraction and Initial Log Compression

For the photographic reproduction methods, it is a typical process to grasp the important information of the image by extracting the luminance channel from the image. To obtain the luminance channel of the image, we convert the input image from the RGB color space to the XYZ color space:

(1) $$\left[\begin{array}{c} X_{in}\\ Y_{in}\\ Z_{in} \end{array}\right]=\left[\begin{array}{ccc} 0.4124 & 0.3576 & 0.1805\\ 0.2126 & 0.7152 & 0.0722\\ 0.0193 & 0.1192 & 0.9505 \end{array}\right]\left[\begin{array}{c} HDR_{R}\\ HDR_{G}\\ HDR_{B} \end{array}\right]$$

where $HDR_{R}$, $HDR_{G}$, and $HDR_{B}$ represent the three RGB channels of the input HDR image. After the matrix transformation in Equation (1), $X_{in}$, $Y_{in}$, and $Z_{in}$ represent the input XYZ channels, where $Y_{in}$ contains the luminance information of the input image. Since human perception of brightness involves a non-linear logarithmic relationship, we then apply log compression to $Y_{in}$ and define the logarithmic luminance $\left(Y_{log}\right)$ as:

(2) $$Y_{log}\left(i,j\right)=\;\log\left(L_{Y}\left(i,j\right)+\varepsilon_{1}\right)$$

where i and j are the coordinates of the pixels in the image. A minimum value $\varepsilon_{1}$ (set at $10^{-6}$ empirically in this study) is added in Equation (2) to avoid the singular value during the compression process.

### 3.2. Pre-Processing for Detail Enhancement

Normally, the local contrast in bright and dark areas tends to be compressed and damaged severely during the reproduction process from HDR to LDR images. To address this problem, this study adopted a detail injection technique that contained two phases. In the first phase, three spatial filters with different radii are used to obtain multiscale feature information. In the second phase, a model of Stevens effects [25] is integrated into our system to fully consider the correlation between each brightness level and its corresponding perceived contrast.

**Figure 3:**
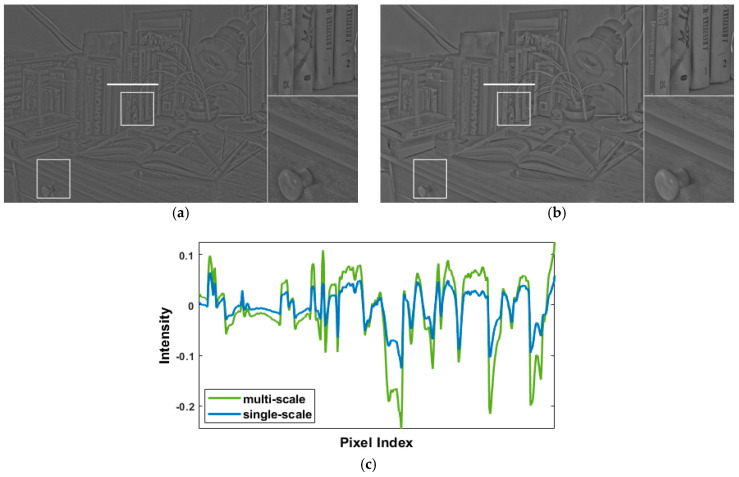
Comparison of the effect between single-scale and multiscale detail extraction using the test image Cadik_Desk02 shown in Section 4.2 (**a**) Result of single-scale-based detail extraction (performed in [24]). (**b**) Result of multiscale-based extraction (performed in the proposed method). (**c**) The pixel value of the detail plane along the horizontal white line segments on (**a**) and (**b**). In (**a**) and (**b**), the right-side images are the enlarged version of the white rectangles, which are suggested to be closely examined by the reader.

As shown in Figure 3a, the detail layer extracted using single-scale decomposition tends to lose multiscale characteristics and is vulnerable to high-frequency noises. To cope with this problem, we adopted a weighted guided image filter (WGIF) [24], an edge-preserving smoothing technique that is robust against halo artifacts, to obtain multiscale features. Two WGIFs with different radii were used: the one with a smaller radius ($r_{1}$) is used for extracting micro-detail features and the one with a larger radius ($r_{2}$) is used for extracting macro-detail features. The procedure of micro- and macro-detail extraction is given by:

(3) $$B\left(i,j\right)=WGIF\left(Y_{log}\left(i,j\right),r_{1},\varepsilon_{2}\right)$$

(4) $$D_{micro}\left(i,j\right)=Y_{log}\left(i,j\right)-B\left(i,j\right)$$

(5) $$D_{macro}\left(i,j\right)=B\left(i,j\right)-WGIF\left(B\left(i,j\right),r_{2},\varepsilon_{2}\right)$$

where B is the base plane, and $\varepsilon_{2}$ is a regularization parameter for penalization. In this work, $r_{1}$, $r_{2}$, and $\varepsilon_{2}$ were empirically set as 15, 30 (double of $r_{1}$), and 0.01.

In Equations (4) and (5), $D_{micro}$ and $D_{macro}$, respectively, indicate the micro-detail plane and the macro-detail plane. The former contains delicate textures such as hair information, and the latter contains structural edges such as outline information of objects. Figure 3b shows the result of merging the micro- and the macro-detail planes. Compared with the single-scale detail extraction (Figure 3a), multiscale micro-and macro-detail extraction (Figure 3b) apparently amplifies more local details and, therefore, relatively avoids the unrealistic visual perception of viewers due to excessive high-frequency noises.

Subsequently, we further apply the concept of the Stevens effect to modify the detailed information. First, the merged detail plane ($D_{merge}$) is defined as:

(6) $$D_{merge}\left(i,j\right)=2\times \left(D_{micro}\left(i,j\right)+D_{macro}\left(i,j\right)\right)-WGIF\left(D_{micro}\left(i,j\right)+D_{macro}\left(i,j\right),r_{3},\varepsilon_{2}\right)$$

Instead of simply adding $D_{micro}$ to $D_{macro}$, the third WGIF with the smallest radius $r_{3}$ (set as approximately half of $r_{1}$) is used to enhance the tiny textures and to improve the detail visibility of the merged detail plane. The color appearance phenomenon explains how lighting conditions affect human perception and the corresponding psychological state. From psychophysical experiments, despite having the same tristimulus values, human eyes may perceive them as different colors due to the inconsistent lighting conditions. For example, a black-and-white image shows relatively low contrast under low-lighting conditions. By contrast, when the same image is moved to a bright area, the white regions become perceivably (cognitively) brighter, and the black regions become perceivably darker. Therefore, the perceived contrast level substantially increases under a bright-lighting condition.

To consider the color appearance phenomenon, the Stevens effect is applied to obtain the injection detail plane ($D_{inj}$) as:

(7) $$D_{inj}\left(i,j\right)=10^{\tau \left(i,j\right)}$$

(8) $$\tau \left(i,j\right)=D_{merge}\left(i,j\right)\times {\left(0.8+F_{L}\left(i,j\right)\right)}^{0.25}$$

In Equation (7), to emphasize the fineness of intensity variation in detail, the processed detail plane is converted back to a linear domain by a power function. In Equation (8), τ involves the merged detail plane and the luminance-dependent factor ($F_{L}$), which is used to adaptively model the Stevens effect at different luminance levels. The $F_{L}$ value is directly adopted from the previous work [26], and it can be expressed as:

(9) $$F_{L}\left(i,j\right)=0.1\times {\left(L_{A}\left(i,j\right)\right)}^{0.33}\times {\left[1-{\left(\frac{1}{L_{A}\left(i,j\right)+1}\right)}^{4}\right]}^{2}+0.2\times L_{A}\left(i,j\right)\times {\left(L_{A}\left(i,j\right)+1\right)}^{-4}$$

where $L_{A}$ is the luminance of the adapted field. Finally, we combine the injection detail plane and the logarithmic luminance plane as:

(10) $$I_{inj}\left(i,j\right)=Y_{log}\left(i,j\right)+D_{inj}\left(i,j\right)$$

The intensity of $I_{inj}$ is further normalized through the following nor function:

(11) $$I_{inj\_n}\left(i,j\right)=nor\left(I_{inj}\left(i,j\right)\right)=\frac{I_{inj}\left(i,j\right)-min\left(I_{inj}\right)}{max\left(I_{inj}\right)-min\left(I_{inj}\right)}$$

**Figure 4:**
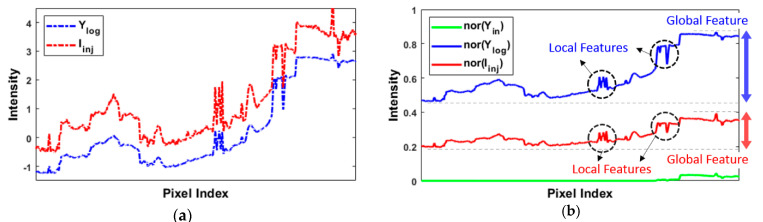
Comparison of the intensity distribution with (and without) the pre-processed detail enhancement along the line segment shown in the bottom right of Figure 1. (**a**) Before normalization. (**b**) After normalization. As shown in the red curve of (**b**), the goal of Section 3.2 was to preserve the local features as much as possible while normally compressing the global features.

Figure 4 shows the pixel intensity distribution in each step; it illustrates the underlying concept of the detail enhancement performed in Section 3.2. As indicated by the green line in Figure 4b, if the luminance channel is directly adjusted by a linear compression, most of the limited dynamic range is preferentially assigned to the regions where local contrasts are relatively high; by contrast, the remaining regions are compressed (to almost zero) severely and thus drastically lose details. Therefore, in view of the nonlinearity between the actual brightness and the brightness perceived by human eyes, we first converted the luminance channel into the logarithmic domain (blue dashed curve in Figure 4a). However, although the major coarse details (i.e., large-scale variations) in the image were maintained, the small-scale details tended to be lost after normalized compression.

To address the above problem, we proposed injecting the micro- and macro-detail planes into the logarithmic luminance plane (red dashed curve in Figure 4a). Moreover, the Stevens effect was applied to consider the color appearance phenomenon in which the perceived image contrast varies as the lighting condition changes. Through the detail injection procedure, the local details are strengthened and are thus still visible after normalization as we desired. Nevertheless, the global contrast of $nor\left(I_{inj}\right)$ was sacrificed, as shown in Figure 4b. In the next step, we deal with this problem by using the HVS-based modified histogram equalization.

### 3.3. HVS-Based Modified Histogram Equalization

In the first stage, the proposed method prioritizes preserving local features. However, the dynamic ranges of images are decreased, and thus, the global contrast is low. To solve this problem, in the second stage, we proposed using the property of image histograms and the HVS characteristics to adjust the configuration of the dynamic range by stretching pixel intensities. Therefore, after reallocation, the overall tone appears in a high-contrast state without sacrificing detailed information.

A histogram is a discrete function that counts the total number of pixels at different intensity levels. Therefore, we can use it to read the information contained in the image. For example, a dark image tends to have the most low-intensity pixels, and so the peak of its histogram will appear at a left-side (i.e., lower intensity) level. In another case, pixels in a low-contrast image tend to distribute over close intensity levels, and so a concentrated and narrow histogram will be generated. In addition, traditional histograms usually accumulate m equispaced bin widths to construct the bin edge $Edge_{k}$ with the same spacing:

(12) $$Edge_{k}=\{\begin{array}{cc} min\left(I_{inj\_n}\right), & if\;k=0\\ Edge_{k-1}+\Delta \omega , & if\;k=1,2,\;\ldots ,m \end{array}$$

where $I_{inj\_n}$ indicates the luminance channel after detail injection, $I_{max}$ and $I_{min}$, respectively indicate the maximum and minimum of $I_{inj\_n}$, and $\Delta \omega =\left(I_{max}-I_{min}\right)∕m$ is the equispaced bin width. The parameter m is used for adjusting the total number of quantification levels in the histogram. A larger m value indicates the use of more intensity levels for rendering a high-quality image. By contrast, a smaller m value indicates that lower computation time is required. Under the trade-off between time and quality, the value of m was empirically set as 60. Moreover, assuming an input image is an unknown signal, the probability $P\left(b_{k}\right)$ assigned to each bin can be expressed by:

(13) $$P\left(b_{k}\right)=\frac{N\left(b_{k}\right)}{Q}$$

(14) $$I_{inj\_n}\left(i,j\right)\in b_{k},\;\;\;\;\;if\;Edge_{k-1}\leq I_{inj\_n}\left(i,j\right)<Edge_{k}$$

where $b_{k}$ is the κ-th bin and is defined as the interval between $Edge_{k-1}$ and $Edge_{k}$, that is, $b_{k}=[Edge_{k-1},Edge_{k})$. The bin count $N\left(b_{k}\right)$ is defined as the number of pixels within $b_{k}$, and Q is the total number of pixels in the image.

Traditional histogram equalization uses a uniform bin width to construct a histogram and subsequently perform histogram-based mapping techniques to adjust the dynamic range. However, for those histograms made from a uniform bin width, the bin counts may vary significantly: For the pixels which belong to the bins with large bin counts, there is insufficient space for stretching the pixel intensities to depict the image details. In contrast, for the pixels which belong to the bins with small (sometimes, even equal to zero) bin counts, they occupy too much dynamic range and thus limit the arrangement of the entire contrast scale. Based on this observation, we found that instead of stretching intensities with the fixed equal-spacing bin width, it was better to arrange each bin width according to the image characteristics dynamically.

In this study, two factors were considered to adjust the dynamic range through the reallocation of the histogram configuration. First, the limited dynamic range is assigned to the bins where sufficient pixels actually exist. Second, a psychophysical metric, the just noticeable difference (JND), is used to balance regional contrast and global contrast. Therefore, the bin width is initialized in proportion to $\left(b_{k}\right)$, which can be expressed as:

(15) $$\omega_{k}=N{\left(b_{k}\right)}^{f}$$

where $\omega_{k}$ represents the initial width of the κ-th bin, and f equals one minus the standard deviation of $P\left(b_{k}\right)$. When the probability of pixels appearing at each intensity level is more dispersed (i.e., the standard deviation is large), the difference between bin counts is larger. Moreover, if the gaps between individual bin widths are wide, the dynamic range is mostly occupied by the intensity levels corresponding to great numbers of bin counts; however, if the gaps are small, the differences among individual intensity levels are indistinguishable from each other, leading to the loss of important information about images. Therefore, we set Equation (15) as a power function and determine the degree of compression based on the degree of probability dispersion. That is, the more obvious the dispersion of the histogram, the smaller is the f value used.

Cutting down the bin widths where bin counts are small and reallocating wider bin widths to the bins where bin counts are large can prevent the situation in which most pixels are at certain narrow intervals of the entire dynamic range. Nevertheless, this is not sufficient. Once an image has big patches that consist of similar colors, a large number of pixels with close intensities are assigned to certain bins, and the pixels of these bins also dominate the dynamic range of the output image, thereby limiting the stretch range of other pixels. Therefore, from the aspect of perceived brightness, we further use the characteristics of HVS to establish a mechanism for correcting $\omega_{k}$.

The background luminance affects the perception of human eyes. The JND metric represents such characteristic of the HVS, which describes the minimum luminance difference between the target and background to be noticeable by human eyes: At the beginning of the experiment, the observers fixate on a screen until they are adapted to the background luminance level (hereafter called the adaptation level, $L_{a}$). Then, the screen starts flashing a disc-shaped light spot, and the observers are asked to report whether the target disc can be recognized from the background. The experiment defines JNDs under different adaptation levels by adjusting the luminance, and as a result, the threshold versus intensity (TVI) curve can be obtained by combining the relationship between the detection threshold and the background luminance in the logarithmic domain. In this study, we directly adopted the JND/TVI model from [27], which can be expressed as:

(16) $$log\left(\Delta L\right)=\left\{ \begin{array}{l} -3.81,\;\;\;\;\;if\;log\left(L_{a}\right)<-3.94\\ {\left(0.405\times log\left(L_{a}\right)+1.6\right)}^{2.8}-3.81,\\ \;\;\;\;\;if-3.94\leq log\left(L_{a}\right)<-1.44\\ log\left(L_{a}\right)-1.345,\\ \;\;\;\;\;if-1.44\leq \log\left(L_{a}\right)<-0.0184\\ {\left(0.249\times log\left(L_{a}\right)+0.65\right)}^{2.7}-1.67,\\ \;\;\;\;\;if-0.0184\leq log\left(L_{a}\right)<1.9\\ log\left(L_{a}\right)-2.205,\;\;\;if\;log\left(L_{a}\right)\geq 1.9 \end{array}\right.$$

where ΔL is the threshold value perceived by human eyes at each adaptation level and the units of both ΔL and $L_{a}$ are $cd∕m^{2}$.

As depicted in Equation (16), the JND/TVI model is defined on a log-log domain. Although human eyes can capture a wide range of luminance intensities, actually two types of retinal cells are used in cooperation—the rod cells function in the dim-light condition, and the cone cells function in the well-lit condition. Therefore, the JND value increases as the adaptation level increases, implying that the bins regarding different luminance intensities inherently require different bin widths; that is, the bins at higher intensity levels need more space for stretching. Considering the abovementioned property, this study proposes the model of a JND-based threshold ($T_{k}^{JND}$) to ensure that the limited dynamic range reaches the most effective arrangement:

(17) $$T_{k}^{JND}=\sum^{\;}\omega_{k}\times \frac{\Delta L_{k}}{\sum^{\;}L_{k}}\;$$

where $T_{k}^{JND}$ represents the maximum permissible bin width of the κ-th bin, and $\Delta L_{k}$ represents the threshold value of the κ-th bin from Equation (16). Because JND is proportional to the background luminance, the maximum intensity in the κ-th bin is set as $L_{a}$ for the calculation of the corresponding $\Delta L_{k}$ so that all pixels in the bin are guaranteed to have sufficient stretched space. Moreover, for those bins whose initial bin widths exceed $T_{k}^{JND}$, pixel distortion may occur in the output image because they initially obtain too much stretched space. Therefore, each initial bin width is corrected by:

(18) $$\omega_{k}^{\prime }=\{\begin{array}{cc} \omega_{k}, & if\;\omega_{k}\leq T_{k}^{JND}\;\\ T_{k}^{JND}, & otherwise \end{array}$$

**Figure 5:**
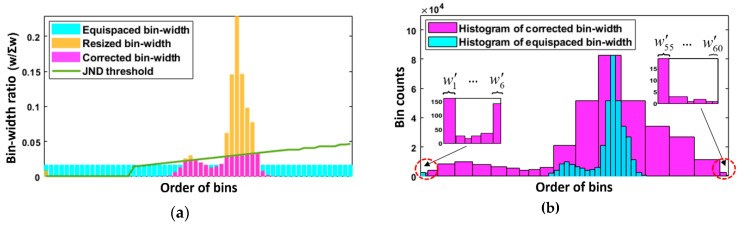
Illustration of the proposed bin width adjustment approach in two aspects. (**a**) Comparison in terms of the output bin width ratios. (**b**) Comparison in terms of the output histograms.

In summary, Figure 5a indicates the variations in bin width ratio arrangement in different stages, where the cyan bars indicate the equispaced bin widths used in the traditional methods, yellow bars indicate the initial bin widths from Equation (15), and magenta bars indicate the corrected bin widths from Equation (18). Considering that if dominant bins (bins with significantly wide bin widths) exist, unnatural colors will occur due to overemphasis of certain pixels, this work utilizes the JND model to define the maximum permissible bin width, i.e., the green curve. As shown in Figure 5a, the bins in which the bin width ratio exceeded their corresponding JND threshold were corrected (i.e., extra bin width is deleted), and the other bins keep their initially allotted bin widths to maintain the relationship of assigning dynamic range to bins that really contain pixels. Figure 5b shows two output histograms. The cyan one was generated using the traditional approach, and the magenta one was generated using the proposed bin width adjustment approach that automatically allocated bin widths and appropriately utilized the dynamic range. Furthermore, the histogram generated using the proposed method not only covers wide intensity levels, which means that the global contrast has been visually expanded, but also helps generate natural tones that are close to the real scene.

### 3.4. Luminance Adaptation and Color Recovery

After bin width adjustment, all bin widths are different from each other, and moreover, all possess a suitable range because both the properties of the HVS and the image content are considered. The limited dynamic range is preferentially assigned to places with abundant details by imposing restrictions on the bins where the probability of pixels appearing is low. Next, the modified bin edges ($Edge_{k}^{\prime }$) can be calculated as:

(19) $$Edge_{k}^{\prime }=\{\begin{array}{cc} 0, & if\;k=0\\ Edge_{k-1}^{\prime }+\frac{\omega_{k}^{\prime }}{\sum^{\;}\omega_{k}^{\prime }}, & if\;k=1,2,\;\ldots ,m \end{array}$$

From the information of the modified bin edges, a look-up table (LUT) is constructed by using the standard histogram equalization method and the linear interpolation scheme. The LUT is used to form the output luminance plane ($Y_{out}$). Because the LUT is a global monotonic mapping function, when rearranging the pixel intensity, artificial artifacts such as blocking and halo effects are guaranteed to be avoided. Finally, the tone-mapped image is obtained as:

(20) $$LDR_{c}\left(i,j\right)={\left(\frac{HDR_{c}\left(i,j\right)}{Y_{in}\left(i,j\right)}\right)}^{s}\cdot Y_{out}\left(i,j\right)$$

where the subscript c∈ {R,G,B} represents the three RGB channels, and s is set as 0.65 to control the saturation.

## 4. Experimental Results and Discussions

### 4.1. Self-Evaluation

To verify the effectiveness of our proposed algorithm, we compared it with five state-of-the-art photographic reproduction algorithms, including a global-based method from [9] (published in 2018), two local-based methods from [11] (published in 2013), and [28] (published in 2020), and two parallel-architecture-type hybrid methods from [21] (published in 2019) and [23] (published in 2017). The test images were obtained from public online resources [29,30,31]. For the comparison of computational performance, taking the image memorial_o876 (with a size of 768 × 512) as an example, the processing time required to generate a reproduced image was 0.6069s (in [9]), 0.9511s (in [11]), 3.8284s (in [21])), 1.3471s (in [23]), 1.0971s (in [28]), and 0.9325s (in the proposed method). All the experiments were performed in MATLAB R2019b with an i7–4790 processor running at 3.60 GHz. In addition to self-evaluation (Section 4.1) of the proposed method, the results of subjective and objective comparison with other methods were also provided in Section 4.2 and Section 4.3, respectively.

**Figure 6:**
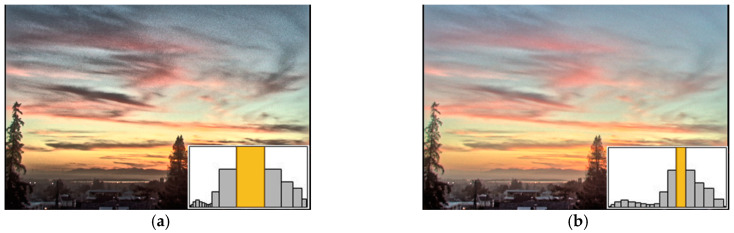
Self-evaluation of the proposed HVS-based modified histogram equalization. (**a**) Result and the histogram before correction, whose bin width is calculated by Equation (15). (**b**) Result and the histogram after correction, whose bin width is calculated by Equation (18). The histograms of (**a**) are too wide so that the resulting image is slightly noisy in the sky.

First, we evaluated the most important property in this study, namely, the HVS-based modified histogram equalization approach. Unlike other methods that simply perform global compression, we proposed the use of a bin width adjustment scheme (and the corresponding histogram equalization) to reallocate the overall tone into a fixed dynamic range. Figure 6a,b show the histograms and the results before and after bin width correction, respectively, where the largest bin widths of each histogram are marked in yellow. In Figure 6a, a large number of pixels have similar luminance intensity; therefore, the yellow bin initially possesses a large proportion of the dynamic range. However, if too much dynamic range is allocated to the pixels with close intensities, the image contrast will be over-stretched and will thus over-amplify some noises, as shown in the sky in Figure 6a. To address this problem, we refer to the characteristic of the HVS and use the JND-based threshold to automatically correct the bin widths that will take up too much dynamic range. As shown in Figure 6b, after bin width correction, the global contrast was maintained, and the output result has a more natural appearance.

**Figure 7:**
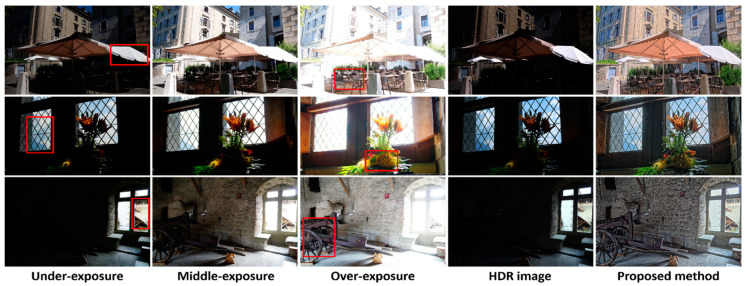
Comparison of multiple-exposure images and the results of our proposed method. The red rectangles indicate the areas which should be closely examined by the reader.

In Figure 7, we refer to images with different exposure levels (LDR images downloaded from [32]) to evaluate our proposed method from a different aspect. Generally, for comparison among images captured by a common camera, the overall tones of middle-exposed images were visually pleasing and close to the real scenes, whereas under- and over-exposed images clearly show the details of bright and dark areas, respectively. Although high-end HDR cameras can record a wider dynamic range of luminance intensities, considerable detailed information tends to be lost when an HDR image is directly displayed on an LDR monitor (second column from the right). As shown in the rightmost column, the results of our method not only maintain natural tones but also preserve the details of the bright and dark areas.

### 4.2. Subjective Analysis

**Figure 8:**
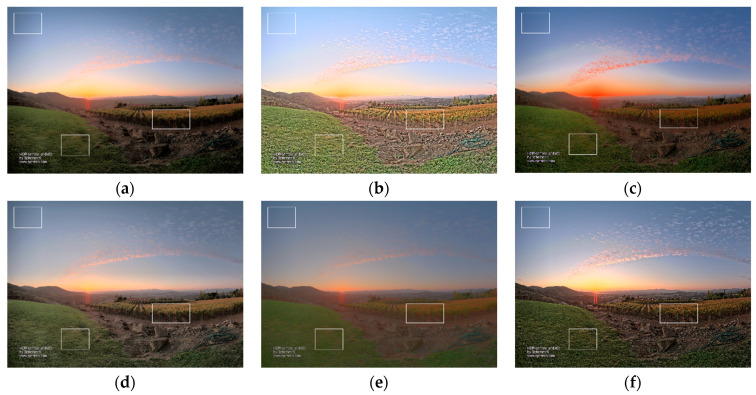
Results of the test image Spheron_NapaValley by (**a**) Khan et al. [9], (**b**) Gu et al. [11], (**c**) Gao et al. [28], (**d**) Ok et al. [23], (**e**) Yang et al. [21], and (**f**) the proposed method. The white rectangles indicate the areas which should be closely examined by the reader.

**Figure 9:**
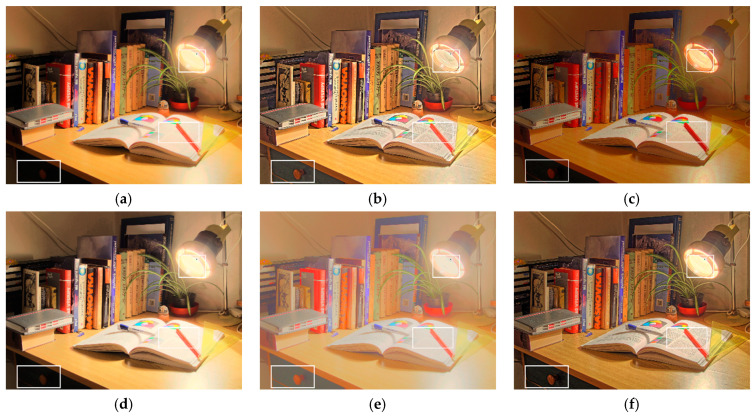
Results of the test image Cadik_Desk02 by (**a**) Khan et al. [9], (**b**) Gu et al. [11], (**c**) Gao et al. [28], (**d**) Ok et al. [23], (**e**) Yang et al. [21], and (**f**) the proposed method. The white rectangles indicate the areas which should be closely examined by the reader.

**Figure 10:**
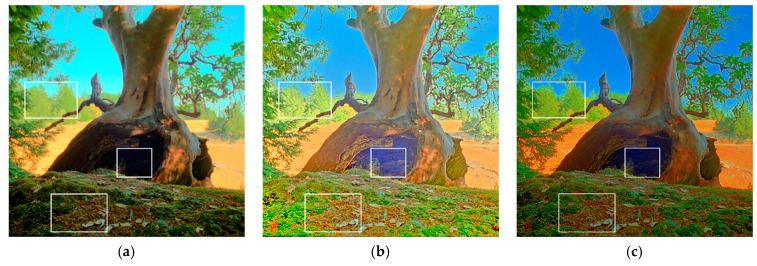

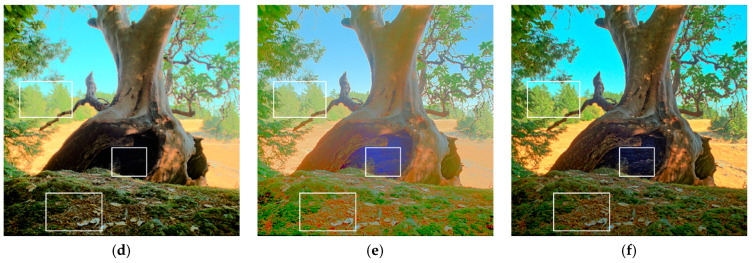
Results of the test image Tree_oAC1 by (**a**) Khan et al. [9], (**b**) Gu et al. [11], (**c**) Gao et al. [28], (**d**) Ok et al. [23], (**e**) Yang et al. [21], and (**f**) the proposed method. The white rectangles indicate the areas which should be closely examined by the reader.

**Figure 11:**
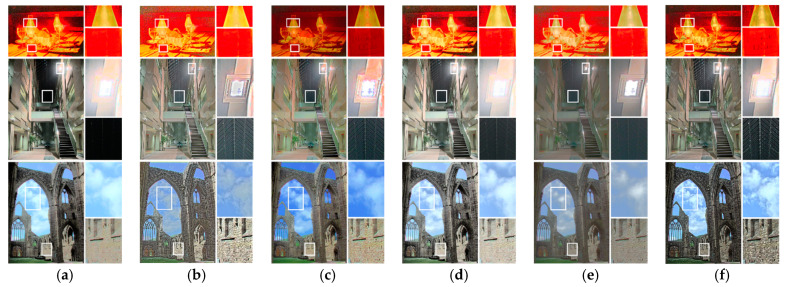
Comparison of the results with close-up images: (**a**) Khan et al. [9], (**b**) Gu et al. [11], (**c**) Gao et al. [28], (**d**) Ok et al. [23], (**e**) Yang et al. [21], and (**f**) the proposed method. The white rectangles indicate the areas which should be closely examined by the reader.

In Figure 8, Figure 9, Figure 10 and Figure 11, we selected images under different conditions to verify whether the proposed method outperforms other methods in having natural tones and rich details. Figure 8 shows the tone-mapped results using the test image Spheron_NapaValley. For Figure 8a,e, although the natural tone of the scene was retained, the details of dark areas can hardly be seen. In Figure 8d, the detail clarity problem was slightly improved; however, the weighted fusion process causes unnatural seams in the sky. In Figure 8b, the details are clearly visible; however, the global tone was faded. In Figure 8c, although the method of [28] improved the problem of detail clarity with contrast; however, the global tone was over-saturated, resulting in a halo effect in the sunset part. The result of our method is presented in Figure 8f, where the trade-off between local and global contrasts was balanced so that it simultaneously retains clear details and the overall color information.

Figure 9 shows the tone-mapped results using the test image Cadik_Desk02. In Figure 9a,d, the global contrast was maintained; however, the detailed information such as the text in the book was lost. In Figure 9b, the details are well preserved; however, artificial artifacts appearing around the lamp were caused by gradient reversal. In Figure 9c, the overall tone is clearly bright, and the details of the text in the book are slightly visible; however, the details in the bottom-left dark area are low. In Figure 9e, an adaptive gamma correction method was used to correct the tones of bright and dark areas separately; however, for a dim indoor scene like this example, an unnatural overall tone tends to be produced. In Figure 9f, the preservation of the natural tone results in a visually pleasing appearance; further, the details are clear, and no artifacts are present because of the use of the proposed multiscale detail injection scheme. Clearly, the proposed method provided the best performance in terms of the coordination of global and local characteristics.

Unlike the indoor scene in Figure 9, Figure 10 shows the reproduced results of an outdoor scene with sufficient lighting: Tree_oAC1. In Figure 10a, the detailed textures of the trunk and the rear trees were not preserved and were thus obscured. In Figure 10b, the sky region and fallen leaves are clear; however, the color is not sufficiently vivid and lacks contrast. In Figure 10c, the details are clear, but the colors are oversaturated, leading to edge distortion, reducing the pleasing visual experience. In Figure 10e, the global chrominance was somehow distorted, and thus, the visual quality was degraded in terms of rendering global tone and local details. Moreover, in Figure 10b,c,e, the noise in the centered tree hole region was amplified. In Figure 10d, although the overall contrast was preserved, the global chrominance was faded (especially in the background). In Figure 10f, in addition to the preservation of naturalness and details, our method prevented high-frequency noise in the tree hole from being amplified and thus provides a visually pleasing appearance.

Figure 11 presents three more examples, with magnified images of the dark and bright areas provided at the right-hand side of each image. An outstanding photographic reproduction method not only maintains the structural information of the input image but also produces natural and attractive results. In terms of structure, the proposed method could effectively preserve the details of bright and dark areas and avoid artificial artifacts that are usually produced by the gradient reversal of local-based photographic reproduction methods. In terms of visual attraction, image components were used to allocate a limited dynamic range dynamically, and furthermore, the characteristics of the HVS were considered. Our resultant images not only conformed to the human visual perception but also provided a good viewing experience for observers.

### 4.3. Objective Analysis

**Figure 12:**
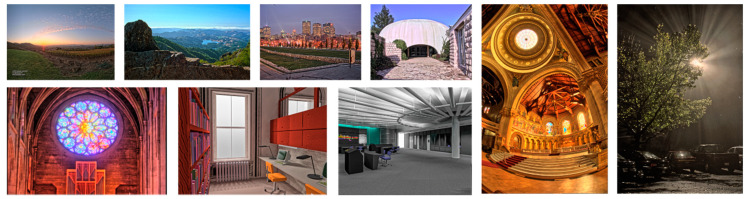
Part of test images from dataset [29]. Images 1 to 4—First row from left to right: SpheronNapaValley_oC5D, MtTamWest_o281, Montreal_float_o935, and dani_synagogue_o367. Images 5 to 7—Second row from left to right: rosette_oC92, rend11_o972, and rend08_o0AF. (Image 8) First from the right: memorial_o876. Image 9: Second from the right: bigFogMap_oDAA. All the images were processed using the proposed method.

In addition to the subjective comparisons, objective evaluation results were obtained using all the images of the dataset in [29], where the dynamic range varied from 2.0 to 8.9, as shown in Table 1. As shown in Figure 12, the images of the dataset from [29] were obtained from various scenes, e.g., outdoor/indoor scenes, day/night scenes, country/urban scenes, and so on. The first objective quality metric is called the tone mapping quality index (TMQI) [33]. It measures the image quality in terms of the structural fidelity (TMQI-S), statistical naturalness (TMQI-N) between the input HDR image and the output LDR result, and overall quality (TMQI-Q) obtained by integrating TMQI-S and TMQI-N by weighted power functions.

Table 2, Table 3 and Table 4 present the TMQI data in terms of TMQI-S, TMQI-N, and TMQI-Q, where the highest and second-highest scores of each row are marked in green and yellow, respectively. The scores of these three evaluation standards are all between 0 and 1. The higher the TMOI score is, the better the image quality of a reproduced image has. Moreover, the total number of the highest scores of each method is counted in the last row, and the one with the highest total number is marked in bold. As shown in Table 2, Table 3 and Table 4, our method has, respectively, 16, 9, and 12 first-ranked images in the three quality indicators, thereby surpassing the other five algorithms in each table. The results listed in Table 2, Table 3 and Table 4 indicate the superiority of the proposed method in terms of different TMQI metrics.

**Figure 13:**
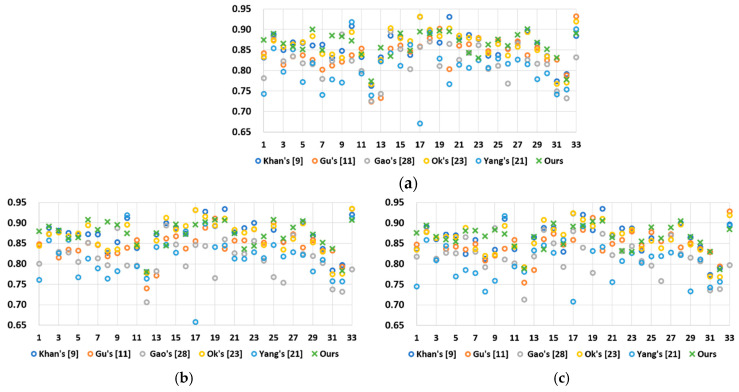
Overall comparison of different methods using the test images in [29]. **(a**) Result of FSITMr_TMQI. (**b**) Result of FSITMg_TMQI. (**c**) Result of FSITMb_TMQI.

The second objective quality metric is called the feature similarity index for tone-mapped images (FSITM_TMQI) [34]. It claims to be an improved version of TMQI because it further considers the phase-derived features. As in TMQI, the score of FSITM_TMQI was between 0 and 1, and a higher one indicates better quality. Figure 13 presents the results of FSITMr_TMQI, FSITMg_TMQI, and FSITMb_TMQI obtained by each method, where the subscript indicates one of the RGB channels. Again, the proposed method exhibited better overall performance than other methods; specifically, it had the top-three scores for most of the 33 images.

The abovementioned indicators are full-reference image quality assessment (FRIQA) techniques that were formulated by referring to the undistorted images. Next, we provide a comparison of two no-reference image quality assessment (NRIQA) techniques: the blind/referenceless image spatial quality evaluator (BRISQUE) [35] and the blind tone-mapped quality index (BTMQI) [36]. BRISQUE refers to the pixel distribution of an image and uses the relationship between normalized luminance coefficients and adjacent pixels to obtain features. BTMQI refers to the analyses of information, statistical naturalness, and structural gradient, which represent different types of features in an image. Both these NRIQA indicators measure the image quality through the features of a tone-mapped image; the lower the score, the better the quality. Regarding the research topic of this paper, as far as we know, the TMQI metrics could be considered the most representative metrics. For example, the TMQI metrics were used in the studies of [9,10,13,18,21,22,23,28,33,34,36]. For the remaining selected metrics, they are commonly used to evaluate the performance of photographic reproduction methods, and they also have been used in many studies. For example, the FSITM metrics were used in the studies of [9,13,33], and the no-reference image quality assessment techniques (BRISQUE or BTMQI) metrics were used in the references of [13,23,28,35,36].

Table 5 presents the results of the averaged score obtained using the abovementioned FRIQA and NRIQA techniques, where the first and second places are marked in green and yellow, respectively. Among the eight objective quality indicators, our method achieved six first-ranked scores and one second-ranked score. Notably, the proposed method ranked only third in TMQI-N. Due to the pre-processing stage of our method, we utilized a detail injection scheme to enhance the local details. The details, especially in the highlight and dark regions, were indeed enhanced and provide visually pleasing results, as shown in Figure 8, Figure 9, Figure 10 and Figure 11; however, the naturalness of the image was affected. Overall, the performance of our work remains remarkable, as shown in Table 5, thereby validating the effectiveness of the proposed method.

## 5. Conclusions

This study proposes a cascaded-architecture-type photographic reproduction method that prioritizes enhancing multiscale local features and then utilizes an HVS-based modified histogram equalization scheme to formulate a global tone adaption curve. Unlike traditional methods that use single-scale decomposition, we used a multiscale micro- and macro-detail injection technique to improve the visibility of local features. Moreover, in parallel-architecture-type hybrid reproduction methods, the final weighted fusion is normally similar to a balance process; to prevent abrupt fusion results, either the clarity of details (advantage of local-based reproduction methods) or the naturalness (advantage of global-based reproduction methods) of tones is sacrificed. As a result, the resulting images from parallel-architecture-type hybrid reproduction methods tend to be vulnerable to dullness. To address this problem, we propose combining the advantages of global-based/local-based approaches in a cascaded architecture to ensure consistency among the dark and the bright regions throughout the image and provide a natural appearance. The experimental results of subjective visual comparisons (Figure 8, Figure 9, Figure 10 and Figure 11) and objective comparisons (Table 2, Table 3, Table 4 and Table 5) validate the effectiveness and superiority of our proposed method.

## Figures and Tables

**Table 1:** List of 33 test images from the dataset from [29] and their dynamic ranges (D).

No.	Name	D	No.	Name	D	No.	Name	D
1	Apartment_float_o15C	4.7	12	StillLife_o7C1	6.1	23	rend04	4.5
2	AtriumNight_oA9D	4.1	13	Tree_oAC1	4.4	24	rend05_o87A	3.3
3	Desk_oBA2	5.2	14	bigFogMap_oDAA	3.6	25	rend06_oB1D	3.6
4	Display1000_float_o446	3.4	15	dani_belgium_oC65	4.1	26	rend07	8.9
5	Montreal_float_o935	3.1	16	dani_cathedral_oBBC	4.1	27	rend08_o0AF	3.7
6	MtTamWest_o281	3.4	17	dani_synagogue_o367	2.0	28	rend09_o2F3	3.9
7	Spheron3	5.8	18	memorial_o876	4.8	29	rend10_oF1C	5.0
8	SpheronNice	4.7	19	nave	6.0	30	rend11_o972	4.1
9	SpheronPriceWestern	2.8	20	rend01_oBA3	3.0	31	rend12	8.9
10	SpheronNapaValley_oC5D	3.2	21	rend02_oC95	4.1	32	rend13_o7B0	4.1
11	SpheronSiggraph2001_oF1E	4.5	22	rend03_oB12	3.2	33	rosette_oC92	4.4

**Table 2:** TMQI-S score of images from the dataset from [29] and the total number of highest scores for each method.

Image	Khan et al. [9]	Gu et al. [11]	Gao et al. [28]	Ok et al. [23]	Yang et al. [21]	Our Method
Apartment_float_o15C	0.8631	0.8132	0.7151	0.8543	0.6364	0.8850
AtriumNight_oA9D	0.9058	0.8809	0.8726	0.8982	0.8928	0.8972
Desk_oBA2	0.8727	0.8526	0.8677	0.8700	0.8146	0.8885
Display1000_float_o446	0.8676	0.8643	0.8325	0.8626	0.8375	0.8925
Montreal_float_o935	0.8344	0.7532	0.7163	0.8416	0.5951	0.8297
MtTamWest_o281	0.9275	0.8758	0.8228	0.8900	0.7278	0.9231
Spheron3	0.8707	0.7543	0.7658	0.8160	0.6619	0.8144
SpheronNice	0.7029	0.6892	0.7090	0.7380	0.5530	0.8004
SpheronPriceWestern	0.8226	0.7663	0.9204	0.8088	0.6084	0.8321
SpheronNapaValley_oC5D	0.9228	0.8802	0.6868	0.9185	0.9226	0.9437
SpheronSiggraph2001_oF1E	0.8086	0.7950	0.6970	0.8329	0.6764	0.8251
StillLife_o7C1	0.7907	0.6368	0.7063	0.7636	0.6164	0.8093
Tree_oAC1	0.8760	0.7691	0.7115	0.8604	0.8512	0.9044
bigFogMap_oDAA	0.9391	0.8468	0.9290	0.9354	0.9058	0.9117
dani_belgium_oC65	0.8974	0.8571	0.8381	0.8763	0.8065	0.8931
dani_cathedral_oBBC	0.8786	0.8775	0.8105	0.8963	0.9014	0.9098
dani_synagogue_o367	0.9735	0.8617	0.7212	0.9677	0.4546	0.9263
memorial_o876	0.8742	0.8575	0.8565	0.8709	0.9061	0.8949
nave	0.8678	0.8276	0.7522	0.8498	0.8632	0.8554
rend01_oBA3	0.8030	0.7738	0.7610	0.7966	0.7197	0.7880
rend02_oC95	0.8930	0.8569	0.8621	0.8772	0.8021	0.8961
rend03_oB12	0.8624	0.8275	0.7643	0.8561	0.8165	0.8443
rend04	0.8763	0.8177	0.8461	0.8619	0.8489	0.8782
rend05_o87A	0.8709	0.7872	0.7453	0.8666	0.7833	0.8730
rend06_oB1D	0.9303	0.9097	0.7550	0.9405	0.9158	0.9210
rend07	0.7663	0.7567	0.6885	0.7513	0.7883	0.7725
rend08_o0AF	0.9016	0.8580	0.8294	0.8799	0.7656	0.9032
rend09_o2F3	0.9246	0.8594	0.7642	0.9138	0.7668	0.8830
rend10_oF1C	0.7600	0.7147	0.6884	0.7459	0.6070	0.7914
rend11_o972	0.8577	0.8156	0.7164	0.8594	0.7961	0.8814
rend12	0.5836	0.6174	0.5058	0.6174	0.5517	0.6533
rend13_o7B0	0.8426	0.7045	0.5385	0.7790	0.5367	0.7138
rosette_oC92	0.8710	0.8782	0.7525	0.8754	0.8743	0.8898
Total number of highest scores	11	0	1	3	2	**16**

Green (and Yellow) numbers indicate the best (and the second-best) performing methods for each row, respectively.

**Table 3:** TMQI-N score of images from the dataset in [29] and total number of highest scores for each method.

Image	Khan et al. [9]	Gu et al. [11]	Gao et al. [28]	Ok et al. [23]	Yang et al. [21]	Our Method
Apartment_float_o15C	0.1924	0.5574	0.3266	0.2325	0.0057	0.5819
AtriumNight_oA9D	0.9231	0.7408	0.9952	0.7588	0.4333	0.8941
Desk_oBA2	0.9384	0.2219	0.3414	0.9309	0.4161	0.8246
Display1000_float_o446	0.9716	0.4511	0.5147	0.8960	0.6954	0.6767
Montreal_float_o935	0.6796	0.5863	0.4827	0.8802	0.0239	0.7591
MtTamWest_o281	0.4447	0.6260	0.7823	0.8542	0.3297	0.9689
Spheron3	0.3273	0.3470	0.2161	0.4647	0.0439	0.6419
SpheronNice	0.2154	0.1818	0.3802	0.3833	0.0275	0.7614
SpheronPriceWestern	0.2384	0.2410	0.6440	0.3327	0.0479	0.8732
SpheronNapaValley_oC5D	0.9703	0.3161	0.2192	0.8007	0.9722	0.3494
SpheronSiggraph2001_oF1E	0.2263	0.7610	0.3065	0.3685	0.0315	0.3866
StillLife_o7C1	0.6606	0.5661	0.2309	0.7384	0.9072	0.5474
Tree_oAC1	0.9983	0.3090	0.7160	0.9311	0.6864	0.8580
bigFogMap_oDAA	0.5713	0.5591	0.5973	0.8172	0.0593	0.1685
dani_belgium_oC65	0.9092	0.8164	0.4998	0.8853	0.3094	0.9810
dani_cathedral_oBBC	0.8907	0.4703	0.2326	0.9974	0.7204	0.6609
dani_synagogue_o367	0.6486	0.3386	0.7619	0.7761	0.1128	0.5031
memorial_o876	0.8038	0.2666	0.1767	0.5786	0.2629	0.4157
nave	0.8101	0.9358	0.1432	0.8677	0.2235	0.9027
rend01_oBA3	0.9663	0.0343	0.3516	0.8414	0.1096	0.6820
rend02_oC95	0.8984	0.7131	0.2555	0.9860	0.2098	0.7876
rend03_oB12	0.9457	0.7657	0.5551	0.9428	0.1120	0.4781
rend04	0.9364	0.7112	0.4196	0.8794	0.1153	0.2038
rend05_o87A	0.5791	0.9096	0.4179	0.6624	0.3368	0.7149
rend06_oB1D	0.3676	0.8107	0.0208	0.5004	0.0254	0.8606
rend07	0.9799	0.8005	0.1296	0.8766	0.3345	0.9331
rend08_o0AF	0.8867	0.9878	0.8208	0.9087	0.4212	0.9290
rend09_o2F3	0.7016	0.2394	0.2692	0.7260	0.0892	0.8307
rend10_oF1C	0.9318	0.9896	0.4474	0.9447	0.1939	0.9357
rend11_o972	0.8177	0.7983	0.6267	0.7643	0.4156	0.8490
rend12	0.0845	0.6519	0.0758	0.1048	0.0364	0.4317
rend13_o7B0	0.1980	0.2979	0.1393	0.2174	0.0635	0.2142
rosette_oC92	0.8591	0.8581	0.1490	0.9440	0.7351	0.5430
Total number of highest scores	8	7	1	6	2	**9**

Green (and Yellow) numbers indicate the best (and the second-best) performing methods for each row, respectively.

**Table 4:** TMQI-Q score of images from the dataset in [29] and total number of highest scores for each method.

Image	Khan et al. [9]	Gu et al. [11]	Gao et al. [28]	Ok et al. [23]	Yang et al. [21]	Our Method
Apartment_float_o15C	0.8279	0.8837	0.8133	0.8344	0.7033	0.9074
AtriumNight_oA9D	0.9653	0.9316	0.9667	0.9389	0.8839	0.9588
Desk_oBA2	0.9587	0.8316	0.8601	0.9569	0.8595	0.9463
Display1000_float_o446	0.9621	0.8795	0.8818	0.9499	0.9127	0.9246
Montreal_float_o935	0.9094	0.8711	0.8424	0.9418	0.6981	0.9204
MtTamWest_o281	0.8950	0.9121	0.9220	0.9511	0.8178	0.9763
Spheron3	0.8582	0.8292	0.8058	0.8685	0.7283	0.8978
SpheronNice	0.8269	0.8113	0.8217	0.8422	0.7117	0.9125
SpheronPriceWestern	0.9764	0.8585	0.9267	0.9505	0.9766	0.9382
SpheronNapaValley_oC5D	0.7866	0.7747	0.7824	0.8311	0.6845	0.8815
SpheronSiggraph2001_oF1E	0.8203	0.9109	0.8038	0.8558	0.7284	0.8570
StillLife_o7C1	0.8941	0.8311	0.7260	0.8984	0.8770	0.8809
Tree_oAC1	0.9681	0.8261	0.8792	0.9543	0.9151	0.9554
bigFogMap_oDAA	0.9197	0.8933	0.9214	0.9574	0.8042	0.8352
dani_belgium_oC65	0.9610	0.9366	0.8808	0.9520	0.8370	0.9702
dani_cathedral_oBBC	0.9534	0.8864	0.8222	0.9733	0.9338	0.9267
dani_synagogue_o367	0.9409	0.8580	0.8892	0.9593	0.6725	0.9049
memorial_o876	0.9393	0.8424	0.8225	0.9031	0.8546	0.8813
nave	0.9386	0.9460	0.7848	0.9422	0.8348	0.9489
rend01_oBA3	0.9434	0.7592	0.8320	0.9235	0.7663	0.8967
rend02_oC95	0.9583	0.9208	0.8414	0.9667	0.8149	0.9427
rend03_oB12	0.9570	0.9208	0.8692	0.9548	0.7954	0.8788
rend04	0.9594	0.9097	0.8689	0.9472	0.8052	0.8345
rend05_o87A	0.9032	0.9308	0.8397	0.9155	0.8357	0.9254
rend06_oB1D	0.8816	0.9498	0.7482	0.9081	0.7947	0.9601
rend07	0.9348	0.9058	0.7617	0.9154	0.8367	0.9299
rend08_o0AF	0.9589	0.9618	0.9297	0.9563	0.8463	0.9654
rend09_o2F3	0.9370	0.8372	0.8166	0.9379	0.7748	0.9457
rend10_oF1C	0.9261	0.9206	0.8275	0.9237	0.7503	0.9357
rend11_o972	0.9370	0.9224	0.8665	0.9294	0.8541	0.9480
rend12	0.7145	0.8385	0.6829	0.7319	0.6874	0.8134
rend13_o7B0	0.8236	0.8044	0.7127	0.8099	0.6910	0.7897
rosette_oC92	0.9467	0.9485	0.7863	0.9602	0.9289	0.9022
Total number of highest scores	9	3	1	7	1	**12**

Green (and Yellow) numbers indicate the best (and the second-best) performing methods for each row, respectively.

**Table 5:** Average score of different objective evaluations for the dataset in [29].

Metric	Khan et al. [9]	Gu et al. [11]	Gao et al. [28]	Ok et al. [23]	Yang et al. [21]	Our Method
TMQI-Q	0.912	0.880	0.834	0.916	0.807	0.912
TMQI-S	0.856	0.807	0.762	0.848	0.752	0.858
TMQI-N	0.684	0.572	0.401	0.721	0.288	0.671
BRISQUE	25.230	25.420	24.283	26.200	26.050	24.100
BTMQI	3.646	3.656	5.010	4.249	4.978	3.202
FSITMr_TMQI	0.860	0.840	0.820	0.860	0.806	0.864
FSITMg_TMQI	0.872	0.848	0.813	0.867	0.816	0.873
FSITMb_TMQI	0.863	0.847	0.819	0.861	0.807	0.865

Green (and Yellow) numbers indicate the best (and the second-best) performing methods for each row, respectively.
